# An example of adaptation: experience of virtual clinical skills circuits of internal medicine students at the Faculty of Medicine, University of Granada (Spain) during the COVID-19 pandemic

**DOI:** 10.1080/10872981.2022.2040191

**Published:** 2022-03-02

**Authors:** Antonio Cárdenas-Cruz, Gerardo Gómez-Moreno, Ana Matas-Lara, Pedro J. Romero-Palacios, Francisco M. Parrilla-Ruiz

**Affiliations:** a Professor, Faculty of Medicine, University of Granada, Granada, Spain; b Student, Faculty of Medicine, University of Granada, Granada, Spain; c Specialist in Family and Community Healthcare, Emergency Service, University Hospital, Granada, Spain

**Keywords:** Home learning, COVID-19, medical student, blended learning, clinical skills circuits, internal medicine

## Abstract

**Background:**

The state of alarm declared in Spain in response to the Coronavirus pandemic (COVID-19) has had far-reaching consequences in all areas of life. At the University of Granada’s (UGR) Faculty of Medicine, online teaching was implemented immediately without any preexisting plan. Second-year undergraduates in medicine, particularly those enrolled in the subject ‘Bases of Internal Medicine,’ would normally undergo clinical skills circuits in face-to-face group settings.

**Objective:**

To facilitate undergraduates’ acquisition of specific transversal skills by means of an integrated online working system.

**Design:**

Before the pandemic, teaching/learning methods consisted of 1) face-to-face group work; 2) teletutoring; 3) written work uploaded to the PRADO online platform for marking by the teletutor; and 4) presentation of written work to the group. As a result of the lockdown, presentations in class were suspended and replaced by online presentations. The means adopted by students in online presentations were freely chosen using various communication techniques: linear projection systems (6); acting/simulation (4); dramatization (1); and role-playing (1).

**Results:**

The number of online clinical skills circuits developed was 12, one for each of the clinical skills circuits established for imparting this subject. A total of 12 presentations were made by the 10 groups, each lasting 15 minutes followed by a 5-minute discussion to settle any questions raised. The presentations were marked jointly by the teaching staff, coordinator, and students.

**Conclusions:**

The transference of classroom learning to the online environment proved an essential resource for teaching/learning clinical/practical skills during the lockdown, which have never before been imparted at distance.

## Introduction

On 14 March 2020, a state of alarm was declared in Spain in response to the Coronavirus pandemic (COVID-19) caused by SARS-CoV-2. This had far-reaching consequences in all areas of life and at the University of Granada, the Faculty of Medicine was forced to implement online teaching/learning almost immediately by order of the university administration. This required a rapid transformation of the teaching/learning process (as was the case in all educational institutions) [1]. In the case of second-year undergraduate medical students taking the ‘Bases of Internal Medicine’ subject, this would normally include the development of clinical skills circuits conducted in face-to-face settings [2,3]. In these classes, a clinical case is analyzed by means of collecting clinical data, examinations, and analyses with subsequent interpretation of the information based on the physiopathological processes that motivated the consultation or caused the main symptom(s). This learning strategy is based on Problem-Based Learning (PBL), found to achieve good outcomes in different fields, and directed at imparting and reinforcing the basic transversal skills that are a key element of the degree course in medicine.

Evidence reported in systematic reviews of online medical training suggests that it is not necessarily less effective than face-to-face learning [4]. In fact, online medical training offers certain advantages and may be considered a complementary teaching method in addition to face-to-face learning. It is also clear that the efficacy of online teaching is influenced by a range of factors that must be kept in mind when it comes to implementing online teaching/learning [4–6].

Empirical evidence suggests that digital education and blended learning are valid equivalents to self-directed and face-to-face classroom/clinical learning for training practicing doctors. George *et al.* [7] and McCutcheon *et al.* [8] argue that the evidence suggests that online learning of clinical skills is no less effective than traditional forms of learning. O’Doherty *et al.* [9] identified a series of obstacles (and the solutions for overcoming them) to online education, which must be recognized when implementing any form of online teaching.

Clinical skills circuits aim to develop generalized critical and deductive thinking in diagnosis. More specifically, their aims are as follows: to facilitate understanding of the processes of interaction between physiopathology and the diagnostic process, to establish direct links between the diagnostic process and semiological elements, to assess efficiency and efficacy of the diagnosis of physiopathology and semiology, and produce a document that establishes a relationship between practical course content and a specific clinical picture. Regarding online experiences of clinical skills circuits in the field of internal medicine among undergraduates, it should be noted that this type of learning has rarely been seen, as this aspect of teaching/learning has traditionally been conducted in face-to-face classroom/clinical situations and fundamentally involves what is known as Objective Structured Clinical Examination (OSCE) [10,11]. This was the prescribed methodology for skills training for second- and third-year students of the Degree in Medicine at UGR.

Recently, blended learning, which integrates online learning and face-to-face classes, has become more relevant in higher education [12,13]. Venkatesh Murthy *et al.* [14] suggest that blended learning could be an effective strategy for teaching/learning as it can successfully integrate the scientific basis with its application in clinical contexts.

The objective of the teaching experience described in this paper was to facilitate specific transversal clinical skills acquisition by implementing an online working system, in which students communicated the conclusions drawn by means of a presentation communicated using methods (simulations, acting out, role-playing, etc.) that they chose themselves, adapted to the ‘home learning’ framework provided by the University of Granada during the COVID-19 pandemic.

## Material and methods

### Experience

The experience reports a specific subject within the syllabus of the second year of the Degree Course in Medicine (Bases of Internal Medicine I, a compulsory subject; official code number: 2221126). This compulsory activity is made up of two compulsory practical assignments, which must be completed in order to pass the subject. The subject is outlined in the course syllabus, approved by the Board of the Department of Medicine, University of Granada, and, therefore, endorsed by the Vice-Rector’s Office for Teaching (29 May 2019). The institution’s human subjects ethical review panel was made up of Prof. José A. Lobón-Hernández (Director of the Department of Medicine, University of Granada), Dr. Amalia González-Jiménez (Secretary of the Department of Medicine, University of Granada), Prof. Pedro J. Romero-Palacios (Coordinator of the subject Bases of Internal Medicine I), and Prof. Antonio Cárdenas-Cruz (Member of the Academic Committee of the Department of Medicine, University of Granada). Informed consent was sought from all students, making them aware that the results obtained would undergo analysis, although this would be entirely anonymous and would not influence the students’ final mark.

**Figure 1.**
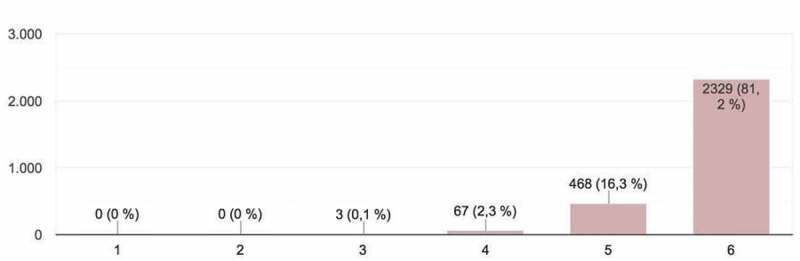
Student evaluations of innovative aspects of the works presented (*Originality*).

The clinical skills circuits were designed by ACC in collaboration with other staff teaching in the Internal Medicine I subject, according to the degree syllabus objectives, which focus on acquiring basic transversal medical skills. This comprises three phases: 1) to establish a relation between physiopathology, semiology, symptomology, and the results of basic complementary exploration, integrating all the information obtained into the diagnostic processes for the major syndromes in medical pathology; 2) to produce a document that explains the relationships between these different entities and processes; and 3) to apply critical analysis to the outcomes and to present the results of the work done to the group. Before the COVID-19 pandemic, the methodology used consisted of 1) group work: groups were set up by the Faculty teaching staff, 12 groups for this particular subject (Figure 1 shows the course schedule); 2) virtual consultation with the teletutor every 15 days via the UGR’s Teaching Resources Support Online Platform (PRADO); 3) production of a document, which is then uploaded to the PRADO platform for marking by the teletutor; and 4) presentation of work to peer group in which the others award a mark based on the quality of the work (giving reasons for the mark decided).

### Adaptation

Over the 3 months that this online teaching/learning activity continued (March–May 2020), the students who took part (almost 100% of those enrolled) adhered strictly to the methodology proposed: they first of all signed a document whereby they committed themselves individually to fulfill the assignment given to each working group/circuit; secondly, a bibliographic search was conducted related to the specific pathological process to be approached; when this search was completed, the findings were uploaded to the teletraining platform for assessment by the teacher responsible for the clinical training circuits. Thirdly, a document was written developing the relationship between semiological data given to the students and the pathological process underlying these data. To complete the assignment, each group prepared and gave a presentation to the students and teaching staff to share the outcomes of the work done. Students were given freedom to choose the format and means of expression for these presentations, which had originally been prepared as group work but were now prepared jointly as an online activity.

Students chose the method of presentation freely, adopting various communication techniques such as stage acting, dramatization, role-playing, and even basic robotic simulation. The evaluation process had two phases. Firstly, the teacher imparting the subject evaluated the quality and design of the document in relation to the clinical case; marks were awarded to each group. Secondly, various teaching staff evaluated individual students’ work in relation to the way in which they presented the clinical case. The evaluation process was continuous, and marks for all learning activities were given to both individual students and the groups. Marks were given for the bibliographic search, document design, and peer group presentation (Table 1).

**Table 1.** Headings for assessment of clinical skills circuits

Group												
			1	2	3	4	5	6	7	8	9	10
1	Mitral stenosis											
2	COPD											
3	Level of consciousness											
4	Syncope											
5	Jaundice											
6	COPD spirometry											
7	Atrial fibrillation											
8	Altered gas exchange											
9	Malnutrition											
10	Anemia syndrome											

*Maximum score: 6*; *Minimum score: 1*

Marking criteria

1. Originality

2. Correct approach

3. Adequate presentation

4. Adaptation to the time allotted

5. Usefulness of the presentation

A total of 270 students participated in the clinical skills circuits (65% women and 35% men), all enrolled in the Bases of Internal Medicine subject for 2019–2020 academic year at the Faculty of Medicine, UGR. They all played an active role in group presentations given from home on 13 May 2020. The online platform used was the University’s SALVE-UGR system Centralized University Videoconference Service using the Zoom® platform (Gartner). This platform will allow access to up to 300 students simultaneously without difficulty and is one of the most widely used videoconferencing resources used by UGR.

## Results

Twelve clinical skill circuits were developed, one for each working group established to impart the subject. A total of 10 peer group presentations were given by the 12 working groups, each lasting 15 minutes followed by a 5-minute discussion and/or resolution of any questions raised. The communication techniques used in the presentations were distributed as follows: linear projection systems (6); stage acting (4); simulation of reality through dramatization (1); and role-playing (1). The topics dealt with by the groups were as follows: mitral stenosis; COPD (physical examination); acute ethanol intoxication; syncope; cholangitis; COPD (spirometry); atrial fibrillation; pneumonia; malnutrition; anemia syndrome; general malaise and fever; and hypovolemic shock.

### Student peer assessment of clinical skills circuits

**Figure 2.**
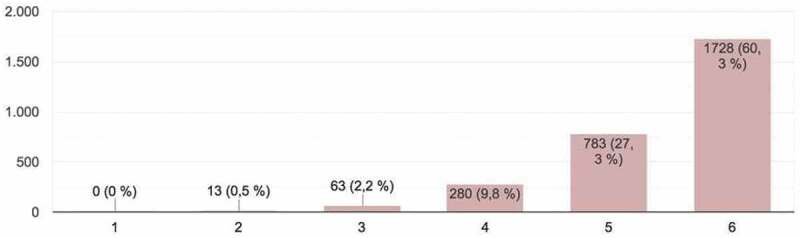
Student evaluations of appropriateness of content to the objectives of clinical skills circuits (*Correct approach*).

**Figure 3.**
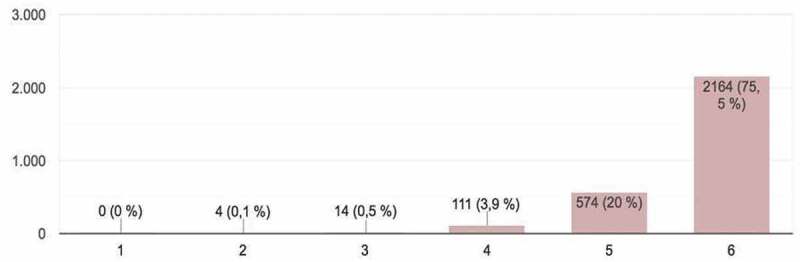
Student evaluations of communication techniques used in presentations (linear presentation, non-linear, stage acting, etc.) (*Adequate presentation*).

**Figure 4.**
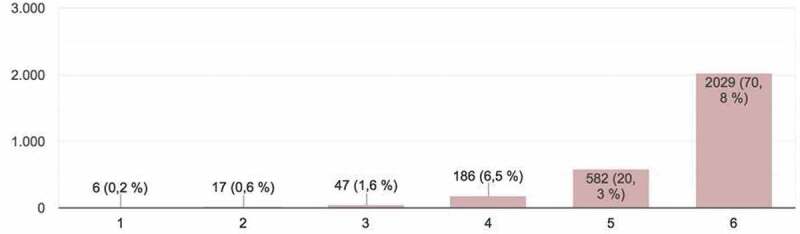
Student evaluation of effective use of time allotted for presentation and discussion (*Adaptation to time allotted*).

**Figure 5.**
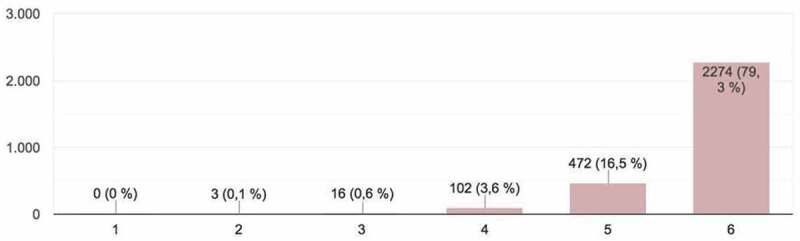
Student evaluation of impact of presentation on future clinical and health-care practices (*Practical usefulness of the presentation content*).

The students provided a total of 2,867 responses, which constituted important input for the development of the activity and could be framed within what is known as ‘hetero-evaluation.’ The parameters evaluated (on a scale of 1 – minimum mark – to 6 – maximum mark) in the online presentations were as follows (Figures 1–5):

1. *Originality*: innovative aspects of the work presented (Figure 1). Most participants in the evaluation process regarded the originality of presentation work as high.

2. *Correct approach*: appropriateness of content to the objectives of each clinical skills circuit (Figure 2). Most participants in the evaluation process expressed how approaches to the topics had been correct, although this percentage was lower than for the originality variable.

3. *Adequate presentation*: communication techniques used in the presentation (linear presentation, non-linear, stage acting, etc.) (Figure 3). Most participants in the evaluation process awarded high marks for the presentations and for the means used to give them.

4. *Adaptation to time* allotted: good use of the full time-span allowed for presentation and discussion (Figure 4). For this variable, although most participants were thought to have used the time allotted well, a statistically insignificant but considerable number of presentations exceeded the time.

5. *Practical usefulness of the presentation content*: the presentation’s impact on future clinical and health-care practices (Figure 5). This variable was particularly important as it focused on the overall usefulness of the work completed by each group and each student.

It should be noted that a significant factor in evaluating group presentations was the fact that these were marked not only by teaching staff and the subject coordinator (denominated super-assessor) but also by each and every participating student, so that the final mark was the sum of marks awarded by teachers, coordinator, and student peers. Fifty percent of the mark was given by students, 40% by teachers, and 10% by the super-assessor for the overall performance of each group over the 3-month evaluation period.

## Discussion

Few examples of this type of teaching/learning experience have been reported that apply to clinical skill circuits in medical training, especially in universities where learning is almost always done in face-to-face classroom/clinical settings. To guarantee the efficacy of online learning, the design principles of digital learning materials, objectives, and the students’ characteristics and preferences must be carefully assessed [4]. In this sense, one of the weaknesses of the present initiative was that we had very little time at the outset to check that the teaching objectives would be met effectively. No information existed from previous years with which the outcomes could be compared because teaching in this subject area had always been imparted in face-to-face classroom/clinical situations. As we had had no previous experience of this kind of teaching/learning, the transformation from face-to-face group activities to the online environment took place suddenly in the middle of the course and developed as it moved forward.

Among the obstacles that affect the online teaching/learning process, O’Doherty *et al*. [9] stress time limitations, insufficient IT skills, inadequate IT infrastructure, inadequate strategies, lack of institutional support, and the potentially negative attitudes of those taking part in the teaching/learning process. In our case, we could rely on support from the UGR from the start, which implemented a home learning program with an integral support strategy for online teaching that involved the entire university and made all the necessary resources available. Throughout the activity, each student had access to a set of didactic resources (video lectures, online simulations, discussion forums, chat rooms, etc.), which helped them to develop both cognitive and procedural skills. In addition, as the culmination of the activity, all students participated in the peer group presentation of the work done using the available resources to create staged simulations or basic robotic simulations. This activity, conducted in the second term of the second year of the degree course in medicine, was completed with another activity carried out during the first term of the third year, which we called ‘poster-discussion sessions.’ In these, the students carried out practices related to the second-year work described in the present article, so that these two activities complemented one another, the second reinforcing the cognitive and procedural skills acquired in the first.

Thanks to the above, each student was able to access and assimilate course content online and make use of the physiopathological resources necessary for their training as future graduates in medicine. Both students and teaching staff had very positive attitudes from the moment that the decision was taken to set up clinical skills circuits in this way. Regarding the students’ IT skills, it was clear that they produced excellent work in the online environment, reflected in the documents produced that demonstrated high levels of scientific understanding expressed in technically well-developed presentations. Although published reviews have concluded that online education needs further investigation in terms of communication between medical students and teaching staff [15], our experience vouches for the online activities developed as an effective means of fulfilling the academic objectives of undergraduate medical training.

For a number of years, the teaching staff on the degree course in medicine have worked towards implementing the concepts of hetero-evaluation [16–19]. This is a teaching resource that involves students directly in peer-group evaluation of their academic activities, always under the guidance of a teacher who has received training in this methodology. Our experiences, as yet unpublished, have focused mainly on postgraduate courses and specialized medical training (of Resident Medical Interns) and has so far achieved very satisfactory results. Regarding assessment outcomes, as the students themselves were involved in the evaluation process (hetero-evaluation), it would be very difficult for them (in the second year of the degree course in medicine) to have acquired sufficient skills at this stage to make a more profound assessment. Nevertheless, all the participants in the evaluation process were provided with specific evaluation criteria/guidelines, which made assessment homogeneous. The criteria applied in the evaluation were as follows: a) originality of the idea; b) approach to the problem to be dealt with; c) development; d) use of appropriate technological resources in the presentation; and e) characteristics of the presentation. This evaluation system allowed us to have a much better-defined idea of the quality of all aspects of the work done by the students.

To our knowledge, hetero-evaluation in undergraduate teaching has not been previously reported. Clearly, it is necessary to develop this in more detail, especially the methodological basis of peer group evaluation. Nevertheless, in this case, student involvement in evaluation showed great strengths, which should be made use of in future and offers much scope for improving students’ learning experience.

## Conclusions

The shift from face-to-face classroom/clinic to online learning has been an essential step toward fulfilling the academic objectives of the UGR’s clinical courses during the COVID-19 lockdown. Clinical skills circuits had never before been conducted at a distance. Despite insufficient time to implement an online learning program and the lack of online resources designed specifically for clinical skills circuits, both teaching staff and students were able to adapt to the new online environment and so meet the expected academic objectives. Online group learning facilitated the teaching/learning process and the acquisition of specific transversal skills required as part of the degree course. It is necessary to have a plan B to fall back on in the immediate future, both in terms of content and resources, in order to respond in an orderly and regulated manner to unforeseen crises such as the current COVID-19 pandemic.
